# O-GlcNAcylation of FoxO1 mediates nucleoside diphosphate kinase B deficiency induced endothelial damage

**DOI:** 10.1038/s41598-018-28892-y

**Published:** 2018-07-12

**Authors:** Shenliang Shan, Anupriya Chatterjee, Yi Qiu, Hans-Peter Hammes, Thomas Wieland, Yuxi Feng

**Affiliations:** 1 Experimental Pharmacology Mannheim (EPM), European Center of Angioscience, Medical Faculty Mannheim, Heidelberg University, Mannheim, Germany; 2 5th Medical Clinic, Medical Faculty Mannheim, Heidelberg University, Mannheim, Germany; 3 DZHK (German Centre for Cardiovascular Research), partner site Heidelberg/Mannheim, Mannheim, Germany

## Abstract

Nucleoside diphosphate kinase B (NDPK-B) acts as a protective factor in the retinal vasculature. NDPK-B deficiency leads to retinal vasoregression mimicking diabetic retinopathy (DR). Angiopoetin 2 (Ang-2), an initiator of retinal vasoregression in DR, is upregulated in NDPK-B deficient retinas and in NDPK-B depleted endothelial cells (ECs) *in vitro*. We therefore investigated the importance of Ang-2 in NDPK-B deficient retinas and characterized the mechanisms of Ang-2 upregulation upon NDPK-B depletion in cultured ECs. The crucial role of retinal Ang-2 in the initiation of vasoregression was verified by crossing NDPK-B deficient with Ang-2 haplodeficient mice. On the molecular level, FoxO1, a transcription factor regulating Ang-2, was upregulated in NDPK-B depleted ECs. Knockdown of FoxO1 abolished the elevation of Ang-2 induced by NDPK-B depletion. Furthermore O-GlcNAcylated FoxO1 was found preferentially in the nucleus. An increased O-GlcNAcylation of FoxO1 was revealed upon NDPK-B depletion. In accordance, the inhibition of protein O-GlcNAcylation normalized NDPK-B depletion induced Ang-2 upregulation. In summary, we demonstrated that the upregulation of Ang-2 upon NDPK-B deficiency is driven by O-GlcNAcylation of FoxO1. Our data provide evidence for a central role of protein O-GlcNAcylation in NDPK-B associated vascular damage and point to the hexosamine pathway as an important target in retinal vasoregression.

## Introduction

Nucleoside diphosphate kinase B (NDPK-B) catalyzes the conversion of nucleoside diphosphates to nucleoside triphosphates^1^. It also regulates numerous cellular activities by forming signaling complexes with other molecules such as calcium-activated potassium channel KCa3.1, Gβγ dimers and caveolin-1^2–7^. Mice lacking NDPK-B have no obvious phenotypes. Development of heart, retinal vessels and T lymphocytes is normal^2,8,9^. Nevertheless, aging mice exhibit decreased heart fractional shortening and sign of retinal vasoregression. T lymphocytes from NDPK-B deficient mice have impaired KCa3.1 channel activity. In pathological conditions, ablation of NDPK-B aggravates catecholamine-induced cardiac remodeling. Most recently, we have identified NDPK-B as a vasoprotective factor. Depletion of NDPK-B impaired angiogenesis in zebrafish embryo model, hindlimb angiogenesis model and hypoxia-induced retinal angiogenesis model, likely by interfering with the plasma membrane distribution of VEGFR-2 and VE-cadherin at the endothelial adherens junctions^9^. Moreover, NDPK-B deficient retinas showed a significant decrease in pericyte coverage and an increase in formation of acellular capillaries mimicking the pathology of early stage diabetic retinopathy (DR). Similar to diabetic wild type retinas, increased angiopoietin 2 (Ang-2) levels were detected in NDPK-B deficient retinas *in vivo* and NDPK-B depleted endothelial cells (ECs) *in vitro*^10^.

The exact mechanism by which diabetes causes retinopathy has been only partially elucidated. The earliest known morphological change in the diabetic retinal vasculature is the loss of pericytes^11–13^. Elevated levels of Ang-2 in the retina are a crucial factor in the induction of pericyte dropout, which finally leads to retinal vasoregression. Our previous data demonstrated that Ang-2 is upregulated in the diabetic retina prior to pericyte dropout^12^. Elevation of retinal Ang-2 by intravitreal injection of recombinant Ang-2 or its overexpression in transgenic mice resulted in a pericyte loss mimicking the DR^12,14^. In accordance, depletion of retinal Ang-2 reduced the hyperglycemia-induced pericyte dropout and formation of acellular capillaries^12,13^.

The expression of Ang-2 is controlled by transcription factors such as mSin3A and FoxO1^15,16^. FoxO1 regulates several cellular processes including cellular differentiation, growth, survival, metabolism, stress response, DNA repair and cell death^17–21^. In endothelial cells, FoxO1 plays an important role for vascular stability through induction of Ang-2. FoxO1 acts downstream of the insulin-dependent signaling pathways, which are dysregulated in diabetes^22^. In diabetic conditions, the modification of FoxO1 by O-linked N-acetylglucosamine (O-GlcNAc) is promoted^23–26^. Uridine diphosphate (UDP)-GlcNAc, the major end product of the hexosamine biosynthesis pathway in glycolysis, is the obligatory substrate of O-GlcNAc transferase (OGT), which catalyzes a reversible post-translational protein modification by transferring O-GlcNAc to proteins^27^. Protein O-GlcNAc modification, like protein phosphorylation, is one of the most common post-translational modifications. It modulates the activities of proteins in nuclei, cytoplasm and mitochondria, regulates gene transcription and protein translation, trafficking and turnover, and contributes to various diseases such as diabetes mellitus, cancer, neurodegenerative and cardiovascular diseases^24,28,29^. Ang-2 expression, for example, is associated with the modification of Sp3 by O-GlcNAc^16^. We established before, that NDPK-B deficiency leads to the elevation of protein O-GlcNAcylation in the retina *in vivo* without the induction of hyperglycemia^10^. We therefore hypothesized that FoxO1, protein O-GlcNAcylation and Ang-2 might be mediators of the endothelial damage induced by NDPK-B deficiency.

We report herein that the increased levels of Ang-2 are indeed crucial for the development of retinal damage under NDPK-B deficiency. Furthermore, we provide evidence that the enhanced O-GlcNAcylation of FoxO1 after NDPK-B depletion is regulating its protein expression and thereby driving the upregulation of Ang-2 in endothelial cells.

## Results

### Ang-2 levels are crucial for pericyte loss and vasoregression in NDPK-B deficient retinas

**Figure 1:**
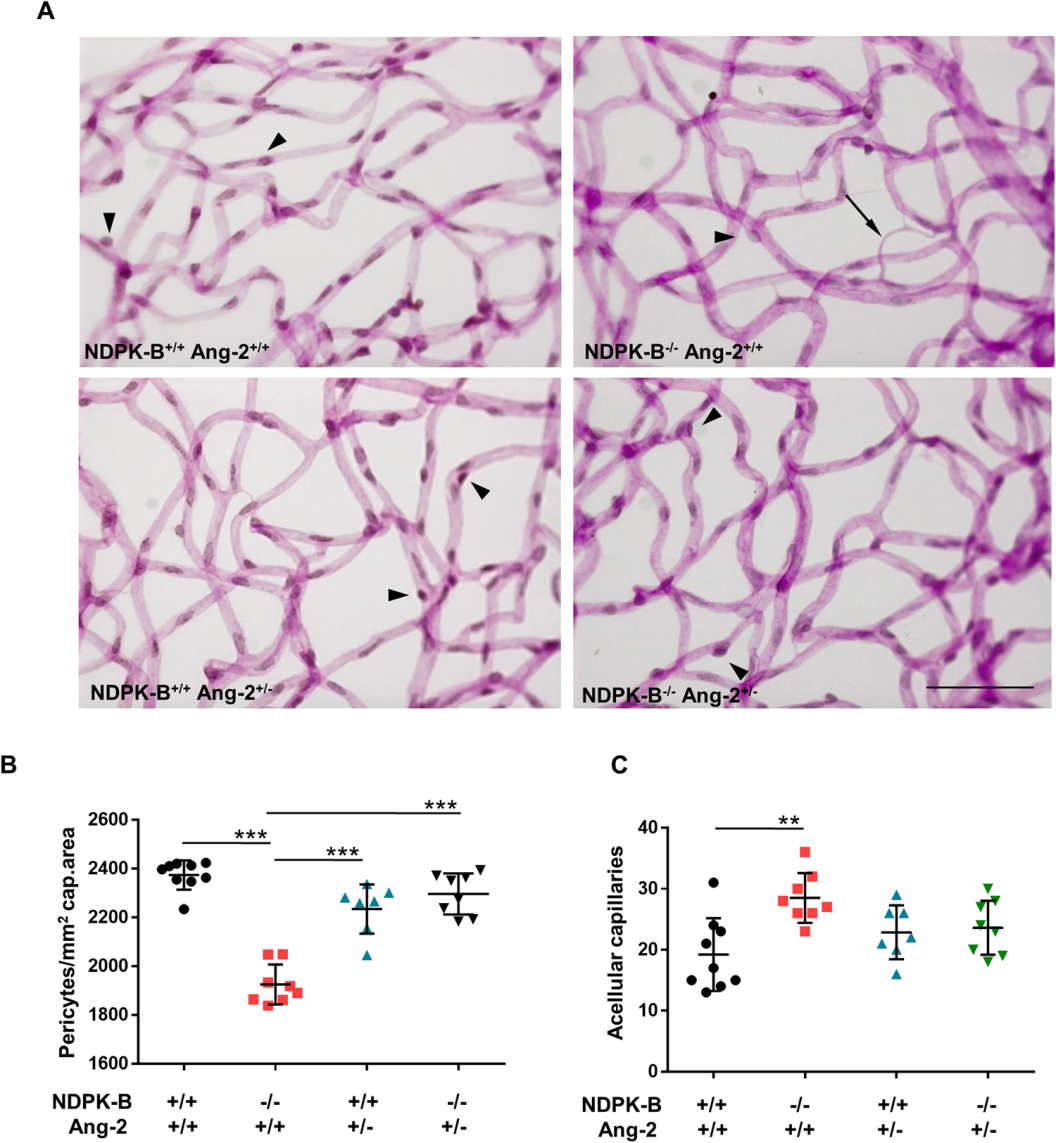
Ang-2 levels are crucial for pericyte loss in NDPK-B deficient retinas. NDPK-B deficiency induced DR-like pathology is rescued by heterozygous loss of Ang-2 in the retinal vasculature. (**A**) Representative examples of retinal digest preparations. Arrowheads indicate pericytes and arrows show acellular capillaries. (**B**) Quantification of pericyte coverage.(**C**) Quantification of acellular capillary segments. n = 7–9, **p < 0.01; ***p < 0.001. Scale bar: 50 μm.

NDPK-B deficient retinas exhibited DR-like pathology which is accompanied by an elevation of Ang-2. To investigate whether upregulation of Ang-2 is responsible for the DR-like pathology observed in NDPK-B deficient retinas, we crossbred the NDPK-B deficient mice with Ang-2 deficient mice to generate NDPK-B^−/−^/Ang-2^+/−^, NDPK-B^−/−^/Ang-2^+/+^, NDPK-B^+/+^/Ang-2^+/−^ and NDPK-B^+/+^/Ang-2^+/+^ mice. NDPK-B deficiency (NDPK-B^−/−^/Ang-2^+/+^) caused significant loss of pericytes in the retinas compared to control mice (NDPK-B^+/+^/Ang-2^+/+^) which confirmed our previously published data^10^ (Fig. 1). Ang-2 haplodeficiency did not yield a significant change in PC coverage in comparison to control retinas. 50% deletion of Ang-2 in NDPK-B^−/−^/Ang-2^+/−^ mice rescued the pericyte loss caused by NDPK-B deficiency in the NDPK-B^−/−^/Ang-2^+/+^ retinas. NDPK-B deficiency also resulted in increased formation of acellular capillaries in the retinas compared to control animals, which correlates with our previously published results. NDPK-B^+/+^Ang-2^+/−^ as well as NDPK-B^−/−^Ang-2^+/−^ retinas showed no significant increase in the number of acellular capillaries in comparison with NDPK-B^+/+^Ang-2^+/+^ retinas. These data verify our hypothesis that the increase in Ang-2 levels is responsible for the DR-like pathology in the retinal vasculature caused by NDPK-B deficiency.

### FoxO1 is upregulated in NDPK-B depleted ECs

**Figure 2:**
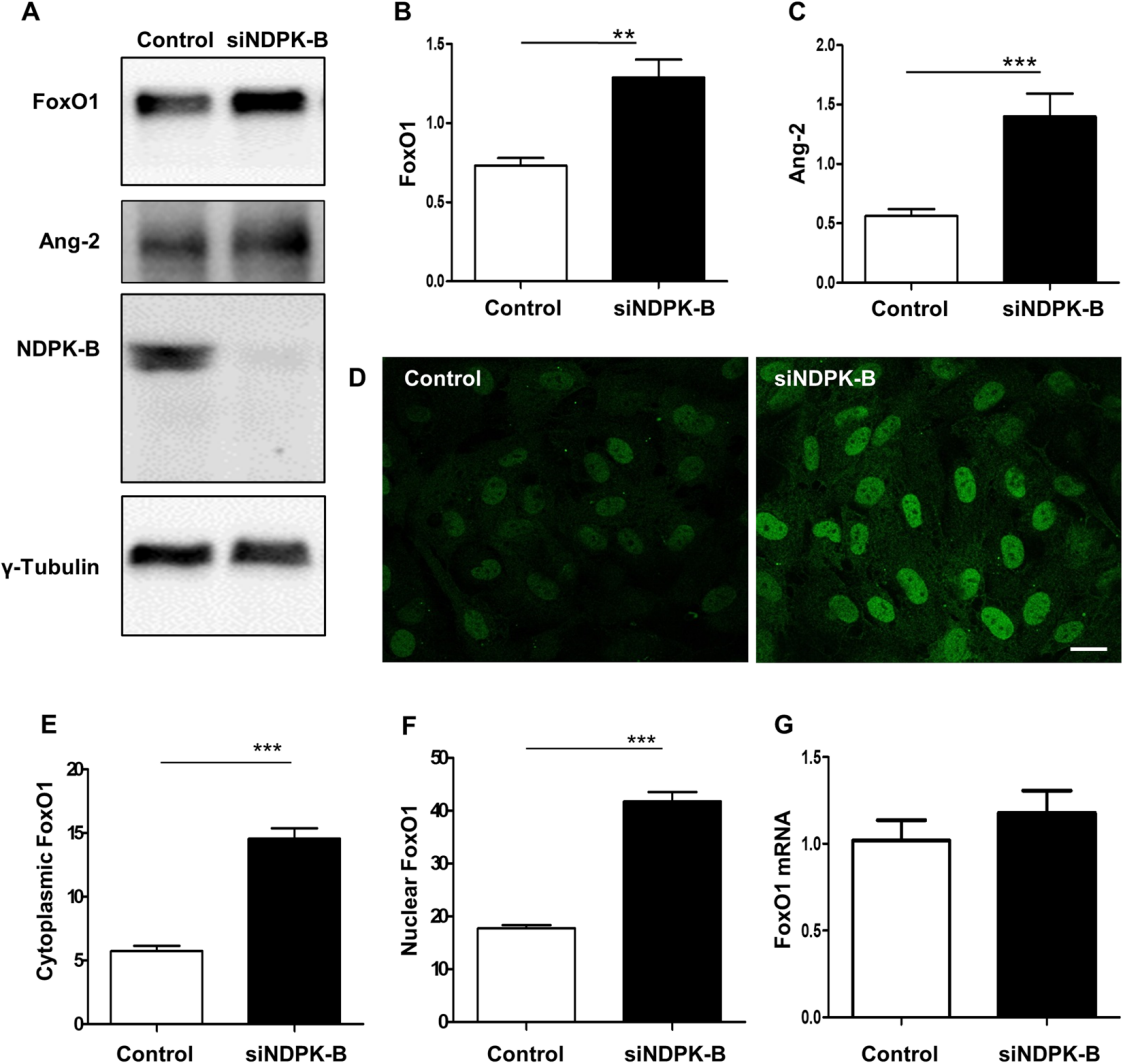
FoxO1 is upregulated in NDPK-B depleted ECs. (**A**) Representative immunoblots of FoxO1, Ang-2 and NDPK-B in NDPK-B depleted ECs. (**B**,**C**) Levels of FoxO1 and Ang-2 normalized to γ-tubulin (n = 7, **p < 0.01, ***p < 0.001). (**D**) Immunofluorescence staining of FoxO1 in NDPK-B depleted ECs (scale bar 10 µm). (**E**,**F**) Quantifications of FoxO1 in the cytoplasm and the nucleus (n = 3). Pixel densities were quantified using the Image J software. Quantitation of the FoxO1 density as obtained from analyzing 20 cells from randomly selected frames for each of three independent EC isolations. (**E**) Transcriptional analysis of FoxO1 by quantitative real-time PCR. The values in control are standardized to 1. n = 4.

FoxO1 is one of the important transcription factors involved in the regulation of Ang-2 expression. Thus, we assessed the expression profile of FoxO1 in NDPK-B depleted ECs. FoxO1 was significantly upregulated in NDPK-B knockdown ECs compared to control transfected cells (p < 0.01, Fig. 2A,B). The upregulation of Ang-2 was also confirmed in NDPK-B depleted ECs (p < 0.001, Fig. 2A,C). To assess the localization of FoxO1, we performed immunofluorescence staining using a specific FoxO1 antibody in NDPK-B depleted and control ECs. In control ECs, FoxO1 was predominantly detected in the nucleus, while a small fraction was detected in the cytoplasm. Upon NDPK-B depletion, increased levels of FoxO1 were observed in the nucleus as well as in the cytoplasm (Fig. 2D). A quantitative assessment by pixel density showed an approximate 200% elevation of FoxO1 in both cellular compartments, the nucleus and the cytoplasm (Fig. 2E,F). We also examined the increase in FoxO1 levels using subcellular fractionation. As shown in Supplementary Fig. S1, we used histone 1 as a nuclear marker and GAPDH as a cytoplasmic marker. Histone 1 and GAPDH are only weakly detectable in the cytoplasmic and nuclear fractions, respectively, indicating successful fractionation. In accordance with data obtained by immunofluorescence staining, nuclear as well as cytoplasmic FoxO1 levels were significantly enhanced in NDPK-B depleted ECs. To study whether NDPK-B depletion induces *foxO1* gene transcription, we quantified the FoxO1 mRNA content up to 24 hr after transfection with NDPK B siRNA by qPCR. As shown in Fig. 2G, no increase in FoxO1 mRNA was detected. These data indicate that NDPK B depletion likely alters the fate of FoxO1 protein, e.g. its posttranslational modification. Nevertheless, the concomitant upregulation of FoxO1 and Ang-2 in NDPK-B depleted ECs points to a role of FoxO1 in the regulation of Ang-2 expression in ECs.

### FoxO1 is required for the upregulation of Ang-2 in ECs after NDPK-B depletion

**Figure 3:**
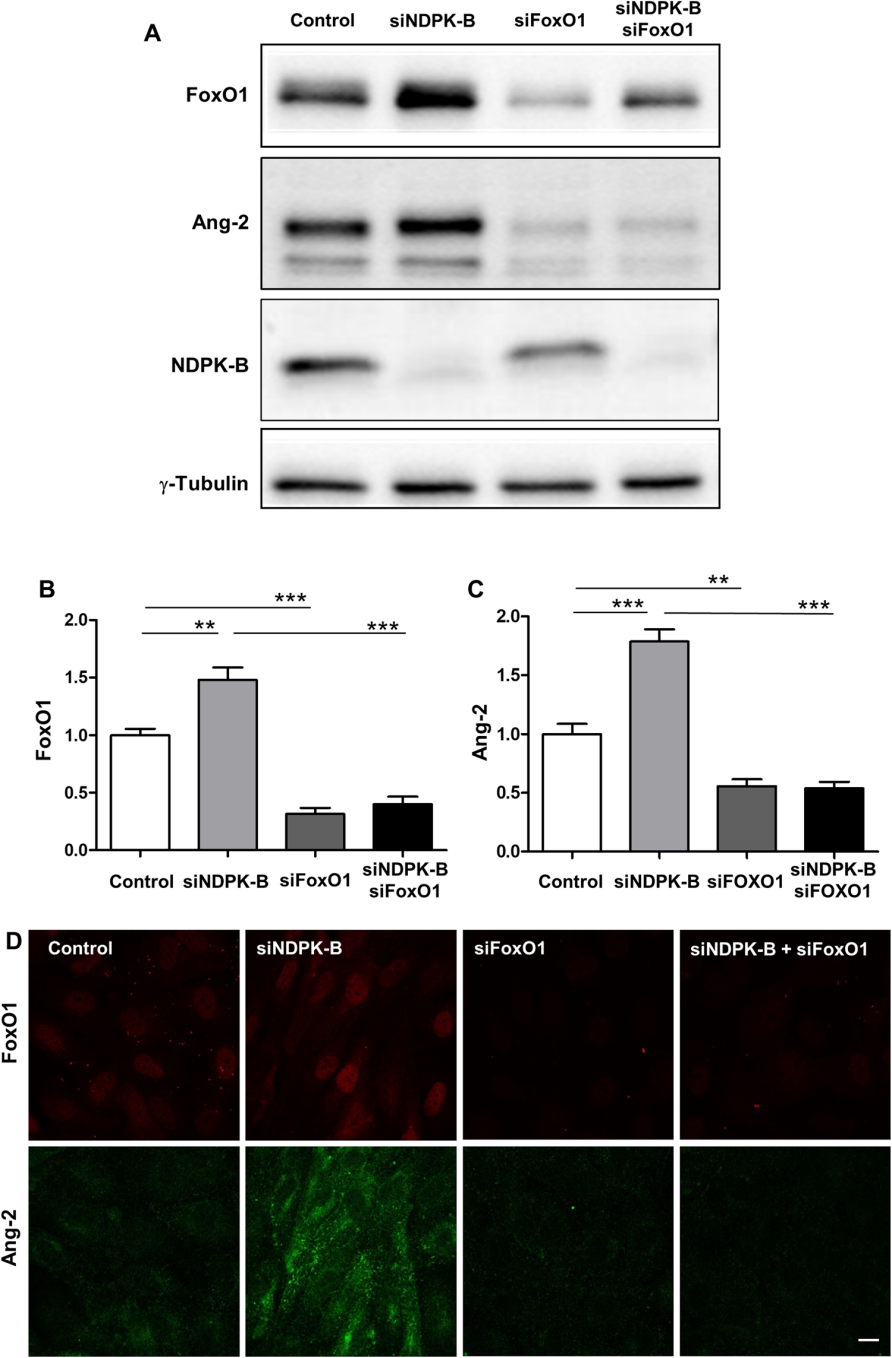
FoxO1 is required for NDPK-B depletion induced upregulation of Ang-2 in ECs. (**A**) Representative immunoblots of FoxO1, Ang-2, NDPK-B. (**B**,**C**) Quantifications of FoxO1 (B) and Ang-2 (C) normalized to γ-tubulin. (n = 7, **p < 0.01, ***p < 0.001). (**D**) Representative images of Immunofluorescence staining of Ang-2 and FoxO1 in FoxO1/NDPK-B single and double knockdown ECs (n = 3, scale bar 10 µm).

In order to prove the importance of FoxO1 for the upregulation of Ang-2 upon NDPK-B depletion, we analyzed the levels of Ang-2 after single and double knockdown of FoxO1 and NDPK-B in ECs. The siRNA-mediated depletion of either FoxO1 or NDPK-B was successfully obtained, showing a 70% and 80% reduction in FoxO1 and NDPK-B levels, respectively (Fig. 3A,B). The quantitative assessment revealed that FoxO1 depletion alone evoked a 50% loss in basal Ang-2 levels (Fig. 3A,C). Furthermore, the consistent upregulation of Ang-2 in NDPK-B depleted ECs was abrogated by FoxO1 depletion (Fig. 3A,C). Immunofluorescence images showed that FoxO1 was successfully depleted both in the nucleus and the cytoplasm. It is well-known that Ang-2 is mainly found in Weibel-Palade bodies in ECs. The expression of Ang-2 in Weibel-Palade bodies was consistently upregulated in NDPK-B depleted ECs, but markedly decreased in FoxO1 depleted ECs with and without NDPK-B knockdown (Fig. 3D). These results indicate that FoxO1 is required for Ang-2 expression and regulation in ECs. The increase in Ang-2 levels upon NDPK-B depletion apparently depends on the elevation of FoxO1 levels. In accordance, the depletion of FoxO1 did not affect NDPK-B levels (Fig. 3A), and Ang-2 knockdown had no influence on FoxO1 expression (Supplementary Fig. S2). Therefore, our data suggest that FoxO1 acts downstream of NDPK-B, and mediates the regulation of Ang-2 expression and its subsequent storage in Weibel-Palade bodies.

### Regulation of FoxO1 by NDPK-B in ECs is independent of Akt or MAPKinase pathways

**Figure 4:**
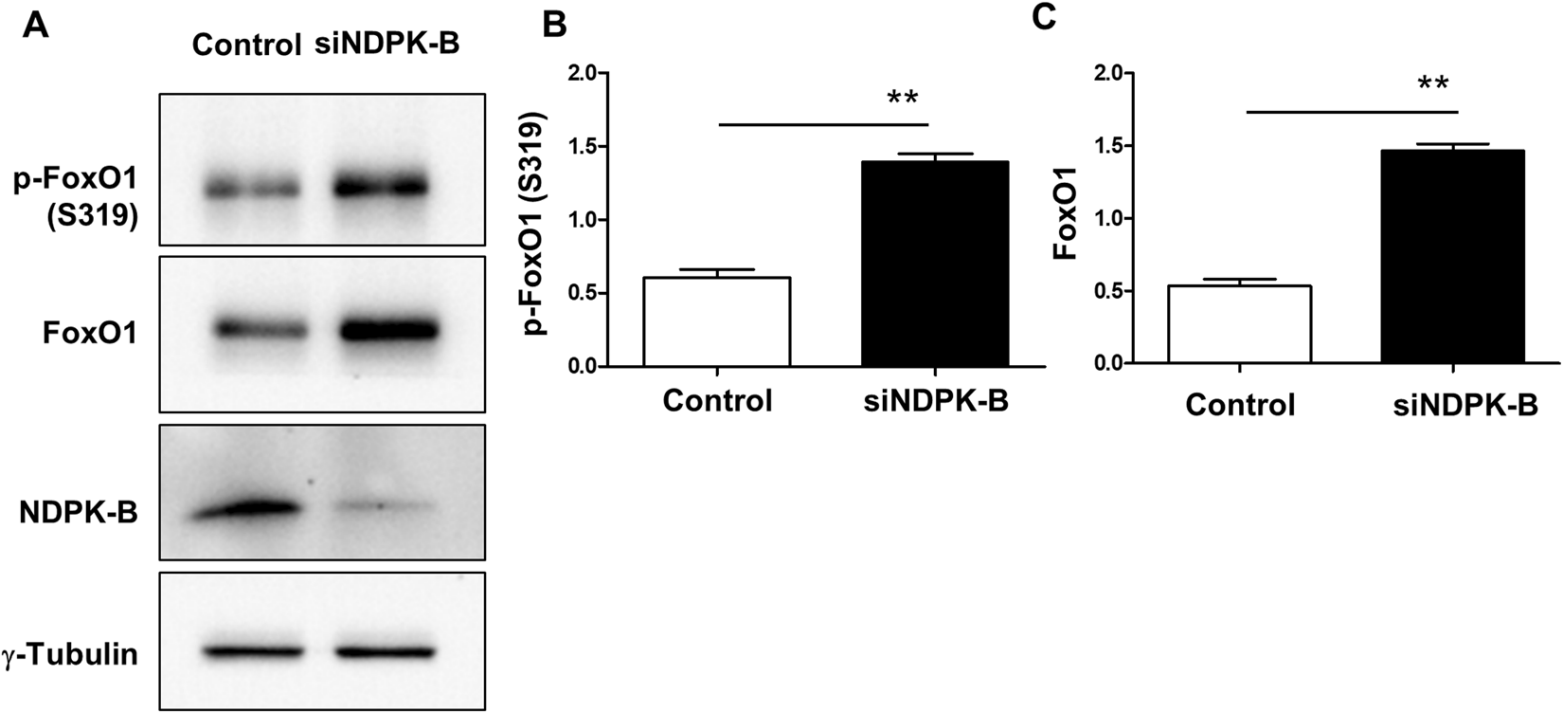
NDPK-B depletion upregulates phosphorylation of FoxO1 in ECs. (**A**) Representative immunoblots of p-FoxO1 (S319), FoxO1 and NDPK-B. (**B**,**C**) Quantifications of p-FoxO1 (S319), FoxO1 normalized to γ-tubulin (n = 5, **p < 0.01).

One of the most prominent possibilities to regulate the activity of FoxO1 is its phosphorylation by the Akt/SGK pathway, leading to the nuclear exclusion and inactivation of FoxO1. In order to test whether this pathway is involved in the regulation of FoxO1 activity upon NDPK-B depletion we explored the status of FoxO1 phosphorylation at its specific target site S319. Comparable to total FoxO1 levels, NDPK-B depletion significantly upregulated p-FoxO1 (S319) levels (Fig. 4A–C). The ratio of p-FoxO1 to total FoxO1 remained unaltered by NDPK-B depletion (control 1.29 ± 0.08, siNDPK-B 1.41 ± 0.10; n = 5, p > 0.05). Moreover, NDPK-B knockdown did not affect phosphorylation of Akt and SGK (Supplementary Fig. S3). FoxO1 is also known to be phosphorylated by the MAPKinase pathway (JNK/c-Jun, p38 and ERK). JNK/c-Jun and p38 activate FoxO1, while ERK, similar to Akt/SGK is able to inactivate FoxO1 via phosphorylation. However, our results showed that NDPK-B depletion did not alter the phosphorylation status and thus activity of JNK, p38, ERK, Akt and SGK1 compared with control transfected cells (Supplementary Fig. S3), suggesting that the regulation of FoxO1 by NDPK-B occurs independent of these kinase pathways.

### NDPK-B depletion enhances O-GlcNAcylation of FoxO1 in ECs

**Figure 5:**
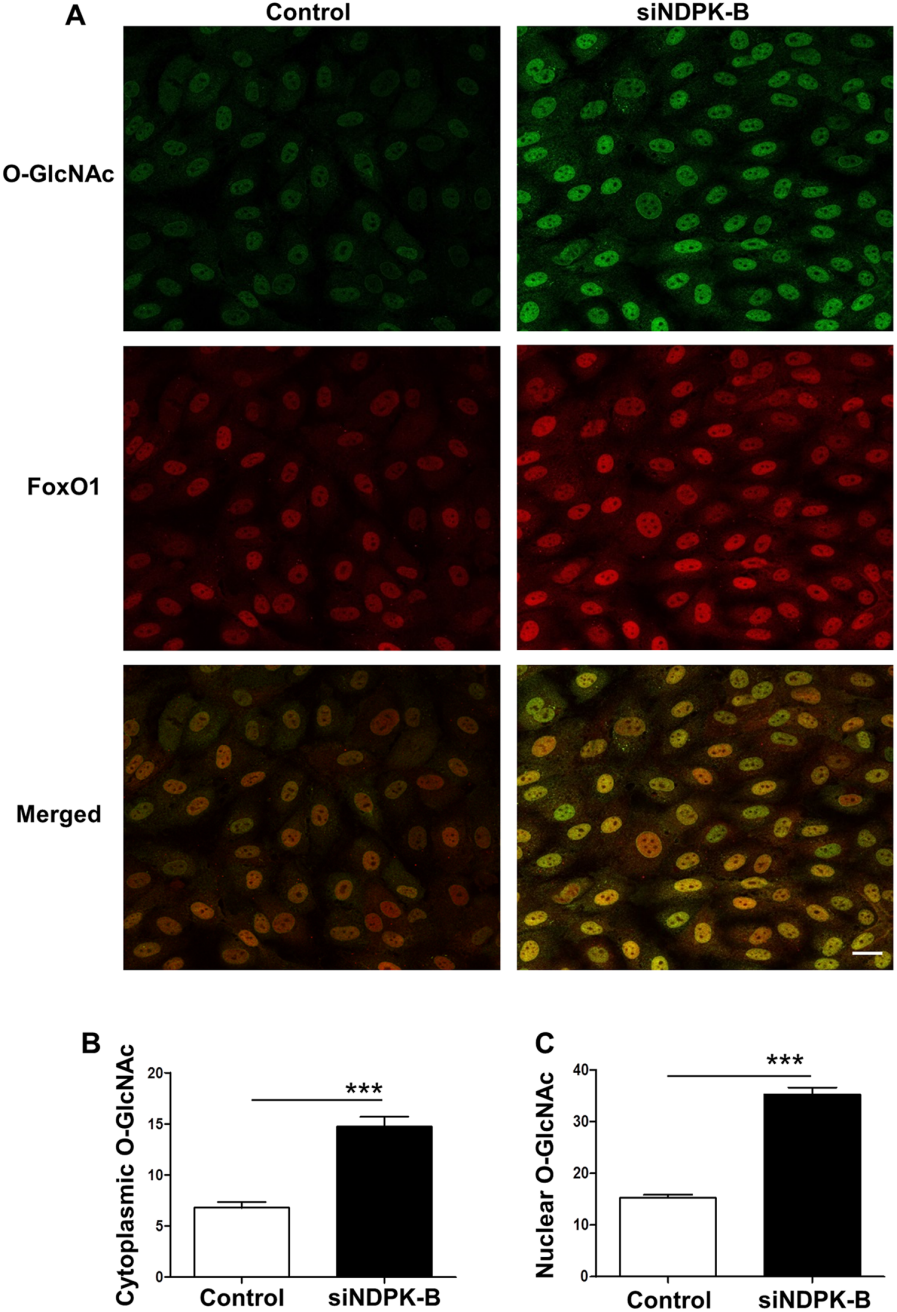
Co-localization of O-GlcNAc and FoxO1 in ECs. (**A**) Representative images show co-localization of O-GlcNAc and FoxO1 in the nucleus and the cytoplasm in NDPK-B depleted ECs. (**B**,**C**) Pixel densities were quantified using the Image J software. Quantitation of the O-GlcNAc density in the cytoplasm (**B**) and the nucleus (**C**) was obtained from analyzing 20 cells from randomly selected frames for each of three independent EC isolations. (n = 3, ***p < 0.0001, scale bar 20 µm).

UDP-GlcNAc generated in the hexosamine pathway by glucose metabolism is able to modify many cytoplasmic and nuclear proteins by covalent modification of serine and threonine residues. Our previous data showed that O-GlcNAc modification of proteins was significantly enhanced in NDPK-B deficient mouse retinas and NDPK-B depleted ECs^10^. As O-GlcNAcylation of FoxO1 has been shown to increase its transcriptional activity^23^, we assessed whether this modification of FoxO1 plays a role in Ang-2 upregulation upon NDPK-B depletion. We used FoxO1 and O-GlcNAc specific antibodies to investigate the localization of FoxO1 and O-GlcNAcylated proteins, respectively. Similar to the FoxO1 expression pattern, O-GlcNAcylated proteins were predominantly found in the nucleus and were weakly detected in the cytoplasm. In accordance with our previous data, the immunofluorescence staining revealed an increased protein O-GlcNAcylation in NDPK-B depleted ECs, concomitant with the upregulation of FoxO1 (Fig. 5A). Quantitative assessment revealed that like FoxO1 levels, protein O-GlcNAcylation was enhanced significantly in the cytoplasm and the nucleus (Fig. 5B,C). As shown in Fig. 5A, the staining of FoxO1 at least partially overlaps with the staining of O-GlcNAc, suggesting FoxO1 is amongst the O-GlcNAcylated proteins.

**Figure 6:**
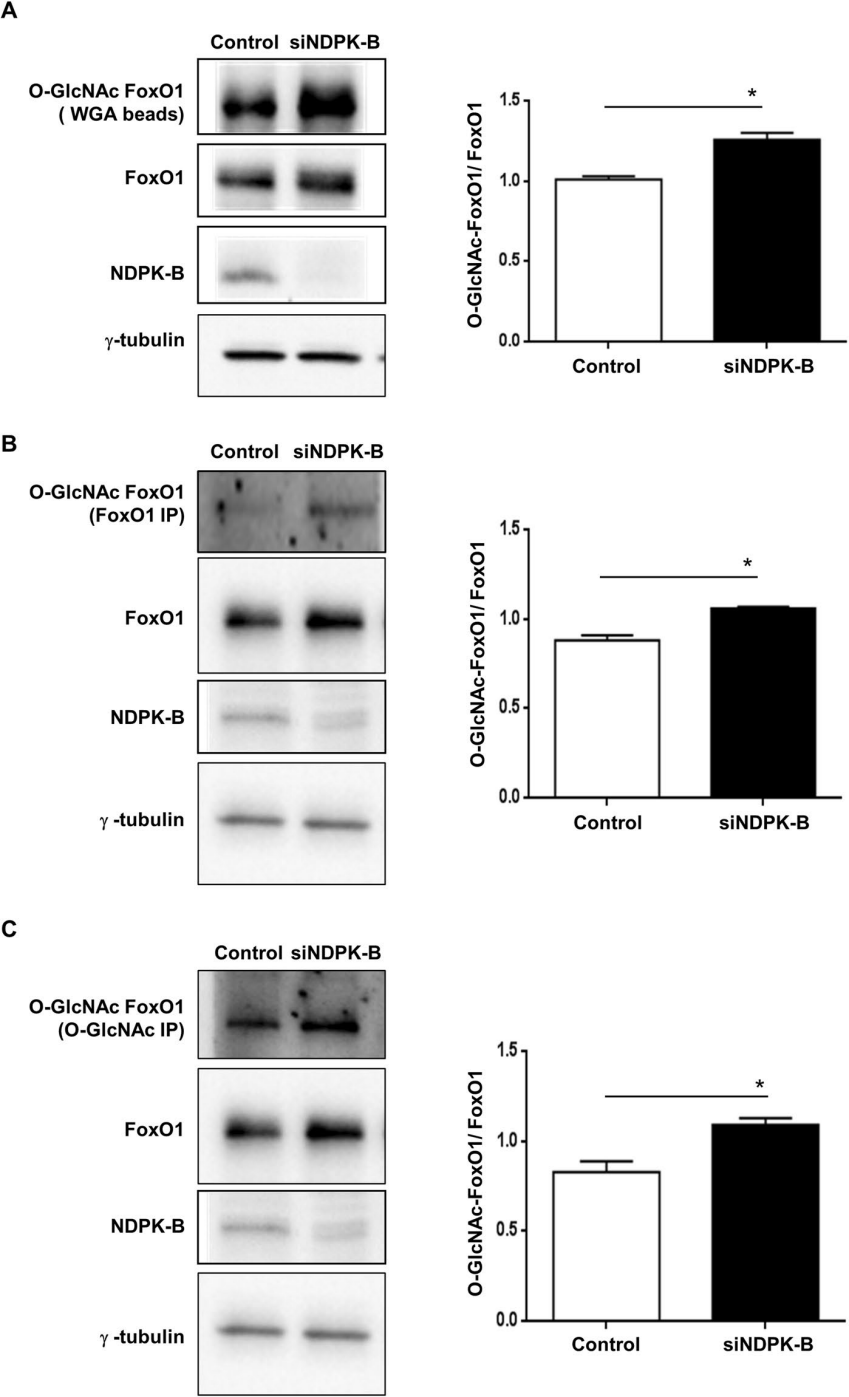
NDPK-B depletion induces increased O-GlcNAcylation of FoxO1 in ECs. Representative immunoblots and quantifications of WGA-agarose beads pull down assay (**A**), FoxO1 immunoprecipitation (**B**) and O-GlcNAc immunoprecipitation (**C**). Whole cell lysates were precipitated with WGA-agarose beads (**A**), or anti-FoxO1 (**B**) or anti-O-GlcNAc (**C**) antibody. The antibodies against O-GlcNAc or FoxO1 were implemented to detect O-GlcNAcylated FoxO1 amongst the precipitated total FoxO1 or O-GlcNAcylated proteins, respectively. The total cell lysates were also probed against FoxO1 antibody to detect total FoxO1. γ-tubulin served as loading control. O-GlcNAcylated FoxO1 was standardized to FoxO1 in the quantifications (n = 3, **p < 0.01).

To prove this hypothesis, we precipitated O-GlcNAcylated proteins with WGA beads and probed for FoxO1 to assess FoxO1 O-GlcNAcylation. As shown in Fig. 6A, WGA precipitation resulted in significant enrichment of O-GlcNAcylated-FoxO1 (standardized to FoxO1) in NDPK-B depleted ECs compared with control transfected cells. To further validate the finding, immunoprecipitations with antibodies specific against O-GlcNAc and FoxO1 were performed. Both immunoprecipitations revealed an enhanced O-GlcNAcylation of FoxO1 in NDPK-B depleted ECs (Fig. 6A,C). Taken together the data show that the O-GlcNAcylation of FoxO1 is elevated upon the loss of NDPK-B and may be responsible for its increased activity, thus augmenting Ang-2 expression.

In order to assess other post-translational modifications of FoxO1, we also immunoprecipitated FoxO1 and investigated for ubiquitination and acetylation. Although acetylation of FoxO1 increased concomitant to FoxO1 levels, the relative acetylation of FoxO1 to total FoxO1 was unaltered. No ubiquitination of FoxO1 was detected in control and NDPK-B depleted HUVECs (Supplementary Fig. S4).

### O-GlcNAcylation inhibitors suppress the NDPK-B depletion-induced upregulation of FoxO1 and Ang-2 in ECs

**Figure 7:**
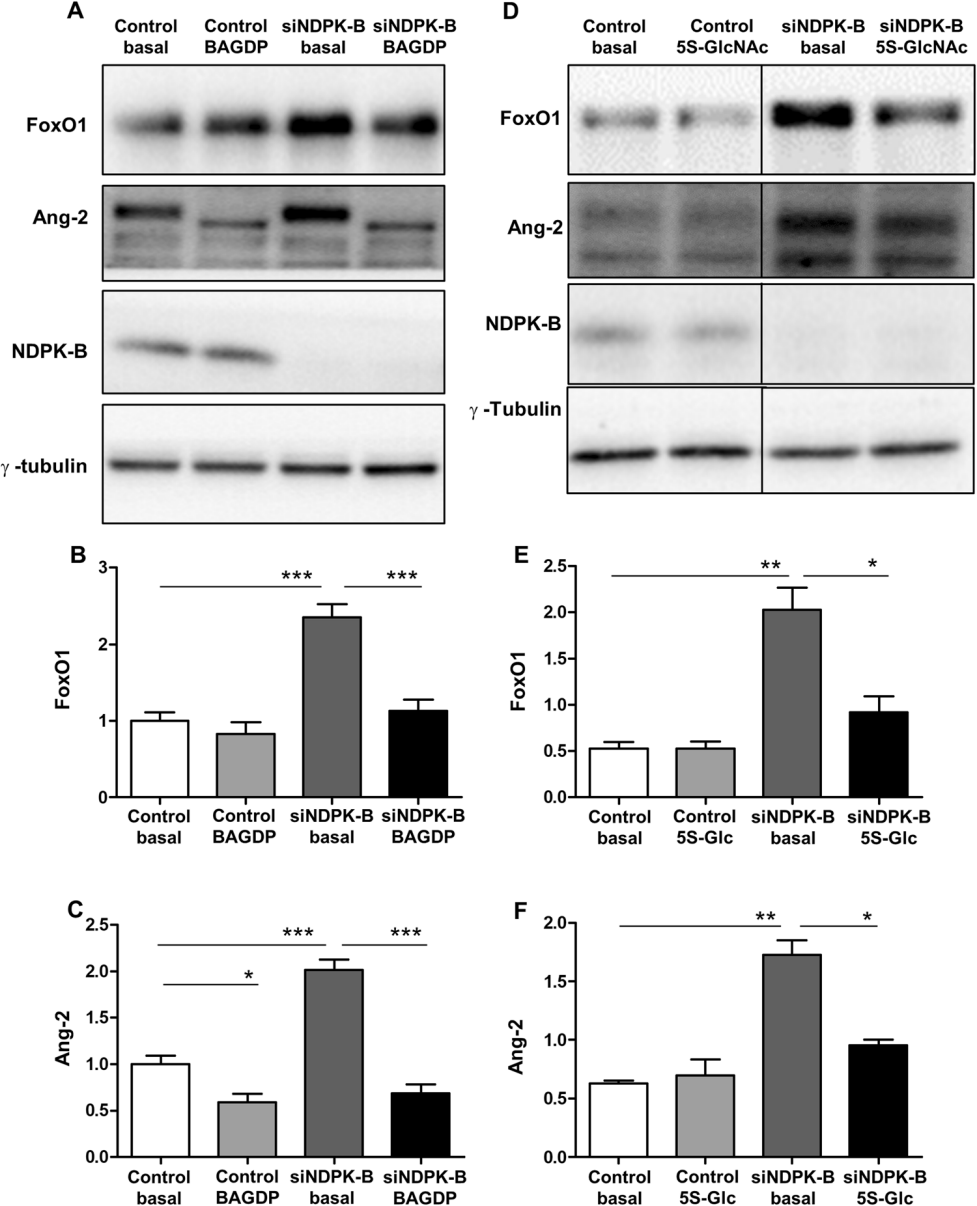
O-Glycosylation inhibitors inhibit upregulation of Ang-2 induced by NDPK-B depletion in ECs. (**A**) NDPK-B deficient ECs were incubated with BADGP or Ac4-5S-GlcNAc. Whole cell lysates were probed with antibodies against O-GlcNAc, NDPK-B and γ-tubulin (loading control) in Western blot. (**A**,**D**) Representative immunoblots of FoxO1 and Ang-2 with BADGP or Ac4-5S-GlcNAc, respectively. (**B**,**E**) Quantifications of FoxO1 in NDPK-B depleted ECs treated with BADGP or Ac4-5S-GlcNAc, respectively. (**C**,**F**) Quantifications of Ang-2 with BADGP or Ac4-5S-GlcNAc, respectively. All of the quantifications were normalized to the respective loading controls. n = 3–5, *p < 0.05, **p < 0.01, ***p < 0.001.

In order to test whether O-GlcNAcylation of FoxO1 contributes to the NDPK-B knockdown-induced increase in Ang-2 levels, we used O-GlcNAcylation inhibitors. We employed Benzyl2-acetamido-2-deoxy-α-D-galactopyranoside (BADGP), which inhibits the OGT mediated formation of O-glycans. Although BADGP did not affect the levels of FoxO1 in ECs, it depressed the expression of Ang-2. Moreover, upon NDPK-B depletion, BADGP treatment prevented the NDPK-B depletion-induced increase in FoxO1 as well as Ang-2 levels (Fig. 7A–C). Surprisingly, BADGP treatment caused an altered migration of Ang-2 protein (Fig. 7A) which appeared to be approximately 8kD in size. As BADGP might inhibit post-translational modifications other than O-GlcNAcylation, which might be important for the cellular processing of Ang-2, we implemented the more specific O-GlcNAcylation inhibitor Ac_4_–5S–GlcNAc (5S-GlcNAc). Compared to BADGP, 5S-GlcNAc had no effect on basal expression of FoxO1 as well as Ang-2 in control cells. Furthermore, 5S-GlcNAc did not induce an obvious alteration in the migratory properties of Ang-2. Similar to BADGP, in NDPK-B knockdown cells, 5S-GlcNAc significantly decreased the NDPK-B depletion-induced upregulation of FoxO1 and Ang-2 (p < 0.05, Fig. 7D–F). Taken together, these data therefore indicate that the increase in FoxO1 levels in NDPK-B depleted ECs is dependent on its O-GlcNAcylation. Moreover, the elevation of Ang-2 was inhibited by both BADGP and 5s-GlcNAc in NDPK-B depleted ECs. It further highlights the importance of FoxO1 in the regulation of Ang-2 under NDPK-B deficiency.

## Discussion

**Figure 8:**
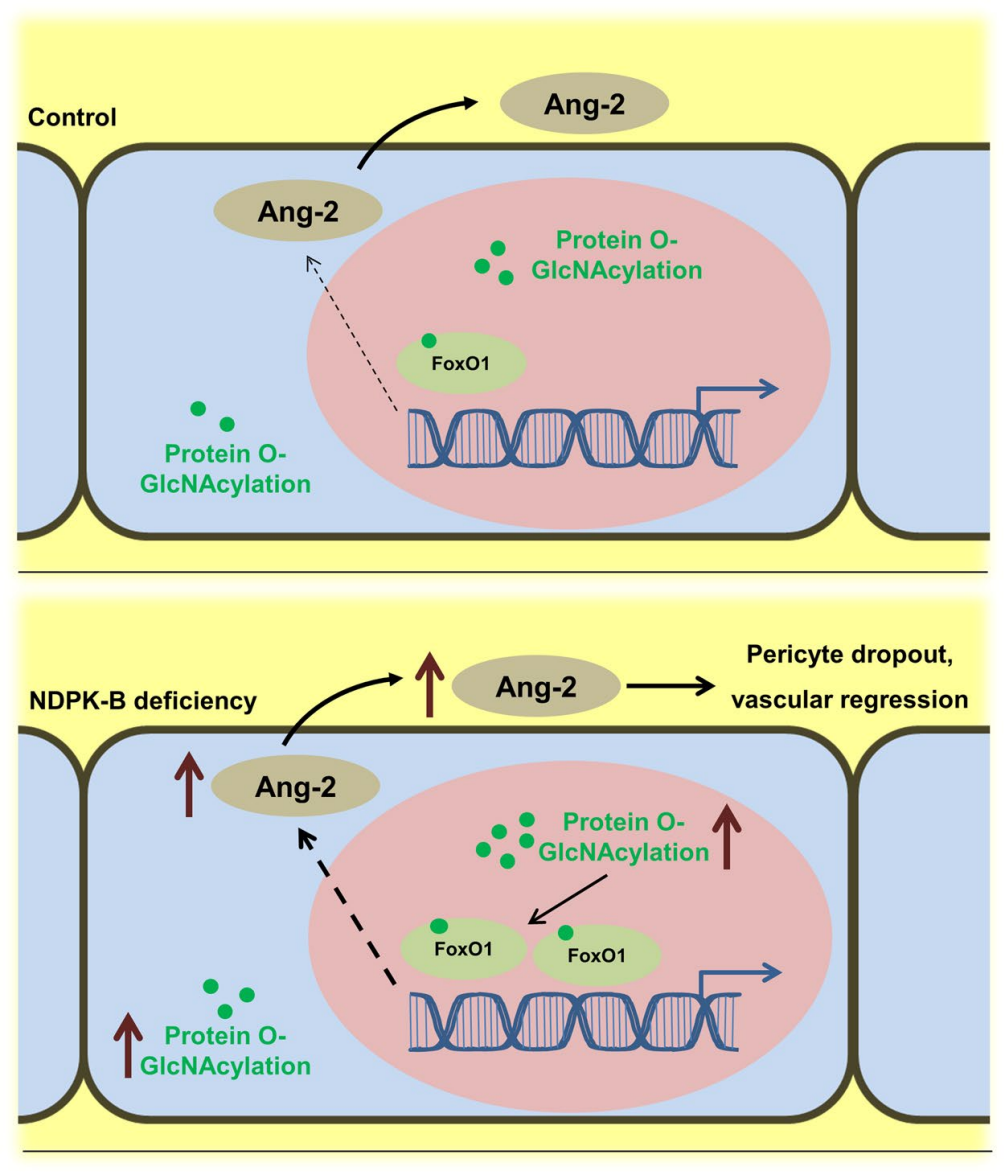
NDPK-B deficiency induces retinal vascular damage through O-GlcNAcylation of FoxO1. NDPK-B deficiency in endothelial cells increases protein O-GlcNAcylation and the amount of the transcription factor FoxO1. O-GlcNAcylated FoxO1 accumulates in the nucleus and thereby increases Ang-2 content and secretion. The increased Ang-2 is driving the loss of pericytes and vascular regression which both are eminent in NDPK-B deficient retinas but not in NDPK-B deficient retinas of mice additionally haplodeficient for Ang-2 (see Fig. 1).

Our study revealed that increased O-GlcNAcylation of FoxO1 and the subsequent upregulation of Ang-2 play a major role in mediating the biological effects of NDPK-B depletion in endothelial cells. Specifically, our study demonstrated that (i) Ang-2 is a key mediator in NDPK-B deficiency mediated vasoregression *in vivo*; (ii) the level of FoxO1 is increased and mediates the Ang-2 upregulation upon NDPK-B depletion in ECs. (iii) the increase in FoxO1 levels upon NDPK-B depletion is dependent on its O-GlcNAcylation and independent of its phosphorylation and subcellular distribution (Fig. 8).

To the best of our knowledge this is the first report to show an increase in FoxO1 levels in ECs after depletion of NDPK-B. FoxO1 is an upstream regulator of Ang-2 in ECs^15^ and has been reported to be involved in enhanced microvascular apoptosis and cell loss in DR^17^ As increased levels of Ang-2 result in pericyte loss and acellular capillary formation in DR^10^ as well as NDPK-B depleted mouse retinas, the increase in FoxO1 in ECs is likely a mediator of vascular response upon NDPK-B depletion. Indeed, the depletion of FoxO1 abrogated the NDPK-B knockdown-induced upregulation of Ang-2. As the depletion of Ang-2 did not alter the protein levels of FoxO1 or NDPK-B in ECs (see supplementary Fig. S2), these data strongly argue for FoxO1 as a mediator of the NDPK-B depletion induced upregulation of Ang-2 in ECs and retinal vessels. The transcriptional activity of FoxO1 is regulated by posttranslational modifications, such as phosphorylation, acetylation and ubiquitination, altering, for example, its subcellular translocation between the nucleus and the cytoplasm^30,31^. Insulin and other growth factors repress FoxO1 activity through activation of the phosphoinositide 3-kinase-Akt/SGK signaling pathway causing FoxO1 phosphorylation, for instance, on Ser319. In contrast, oxidative stress induces FoxO1 activation through multiple posttranslational modifications^32–34^. Interestingly, we found no alteration of the relative phosphorylation of FoxO1 at Ser319 and no indication for an altered activity of known upstream regulators of FoxO1 activity such as Akt/SGK, JNK, p38 and ERK. In line with these data, our immunofluorescence and subcellular fraction experiments demonstrated that depletion of NDPK-B did not alter the subcellular distribution of FoxO1 between the nucleus and the cytoplasm. In accordance, NDPK-B deficiency did not alter FoxO1 ubiquitination or acetylation in ECs (see Supplementary Fig. S4). We show however that the amount of nuclear FoxO1 is significantly elevated due to the increase in its absolute amount. Assuming that nuclear FoxO1 is transcriptionally active, this increase in nuclear FoxO1 is likely causing the increased Ang-2 expression upon NDPK-B depletion in ECs. As mentioned before, in accordance with interpretation, the NDPK-B depletion induced expression of Ang-2 was abrogated by the concomitant knockdown of FoxO1.

FoxO1 is modified and activated in response to glucose by O-GlcNAcylation implicating a role of this covalent modulation and the hexosamine pathway in the regulation of FoxO1 activity^25,26,35^. We reported before that NDPK-B depletion in ECs as well as NDPK-B deficiency in the mouse retina elevated protein O-GlcNAcylation^10^. Interestingly, FoxO1 as well as the O-GlcNAcylated proteins were co-localized in the nucleus, raising the possibility that NDPK-B depletion increases the O-GlcNAcylation of FoxO1. Indeed, the pull down with WGA and the reciprocal immunoprecipitation confirmed the increase in O-GlcNAcylation of FoxO1 in ECs after NDPK-B knockdown. However, the enhanced O-GlcNAcylation of FoxO1 did not influence its relative subcellular distribution, which is consistent with published data^35^. Interestingly, the treatment of ECs with known inhibitors of GlcNAcylation, BAGDP and Ac4-5S-GlcNAc suppressed the NDPK-B depletion-induced increase in FoxO1 levels, which clearly demonstrates that the enhancement of O-GlcNAcylation contributes to the increase in total FoxO1 levels. FoxO1 can be O-GlcNAcylated at residues Ser550, Thr648, Ser654, Thr317 or Ser318. The O-GlcNAc modified sites in FoxO1 in NDPK-B depleted ECs remain unclear. It might be speculated that the modified residue is Thr317 which has been shown to be targeted in high glucose conditions^35^. Protein O-GlcNAc modifications like many phosphorylations occur on serine and threonine residues. Thus, O-GlcNAcylation and phosphorylation can be competing modifications, and it is known that O-GlcNAcylation protects a variety of proteins from ubiqitination and proteasomal degradation^36^. Taking into account that we could not observe an enhanced transcription of FoxO1 upon NDPK-B depletion our data argue for the inhibition of FoxO1 degradation as an underlying cause for the observed increase in FoxO1 levels. Although O-GlcNAcylation of FoxO1 does not alter its subcellular distribution, it increases its absolute amount in the nucleus and thereby obviously drives Ang-2 expression. In line with this hypothesis, BADGP and Ac4-5S-GlcNAc abolished the upregulation of Ang-2 in NDPK-B depleted ECs. Interestingly, BADGP but not Ac4-5S-GlcNAc induced an obvious shift in the migratory properties of Ang-2 in SDS-PAGE. These results are in accordance with published data showing that BADGP, besides being O-GlcNAcylation inhibitor, might also be interfering with N-glycosylation. In contrast, Ac4-5S-GlcNAc appears to be a specific O-GlcNAcylation inhibitor^37^.

Although NDPK-B depletion mimics high glucose, resulting in Ang-2 upregulation followed by endothelial damage *in vivo* and *in vitro*, with respect to FoxO1 regulation, NDPK-B differs from high glucose. NDPK-B depletion induces FoxO1 expression whereas high glucose does not alter its expression. Also, from hyperglycemia models and high glucose treatment, it is able to activate FoxO1 via phosphorylation and O-GlcNAcylation, instead of direct regulation in FoxO1 expression. Our results with O-GlcNAc inhibitors showed for the first time that O-GlcNAcylation directly regulates FoxO1 expression, indicating that FoxO1 expression is O-GlcNAc dependent. In physiological conditions, insulin suppresses FoxO1 activity through Akt phosphorylation signaling, while high glucose elevates FoxO1 O-GlcNAc modification, and subsequent FoxO1 activation. NDPK-B depletion might imitate high glucose, O-GlcNAcylating FoxO1, being independent of FoxO1 phosphorylation, causing DR-like endothelial damage.

We reported before that NDPK-B deficiency leads to a vascular pathology mimicking diabetic retinopathy with regard to vasoregression^10^. Ang-2, which is crucial in the initiation of the retinal vasoregression in DR^13,14^, is also upregulated in NDPK-B deficient retinas. Our results (see Fig. 1) provide evidence that it is the crucial initiator of vasoregression in this model as well. Our finding in the retina, in combination with the data on decreased Ang-2 under O-GlcNAcylation suppression in ECs, supports previous reports showing hexosamine pathway is involved in the development of diabetic vascular damage^38–40^. The main difference between DR and NDPK-B deficiency is that the latter is not associated with hyperglycemia^10^. Nevertheless, a common determining factor is the elevation in protein O-GlcNAcylation as an indicator of an increased activity of the hexosamine pathway. In DR, this increase is driven by hyperglycemia leading to an enhanced glucose uptake and metabolism. Interestingly, the increase in protein O-GlcNAcylation caused by hyperglycemia and NDPK-B deficiency in the retina is not accumulative. Likewise, high glucose treatment of ECs and NDPK-B depletion cause a similar increase in protein O-GlcNAcylation, which again is not accumulative^10^. These data indicate that there is a maximal activation of the hexosamine pathway or the OGT which can be achieved by either pathway. In the NDPK-B deficiency model, this is driving the O-GlcNAcylation and stabilization of FoxO1 leading to enhanced Ang-2 expression. In certain models of hyperglycemia and high glucose treatment of FoxO1 expressing cells, an increase in FoxO1 O-GlcNAcylation and protein levels were reported^35^. Although the alterations of the phosphorylation of FoxO1 might additionally contribute to its altered activity, our data indicate that its O-GlcNAcylation and the subsequent enhancement of Ang-2 is of major importance for the pathology of DR. This also argues that inhibition of O-GlcNAcylation is a feasible therapeutic intervention target to prevent retinal vasoregression.

The cause of elevated protein O-GlcNAcylation upon NDPK-B depletion remains unknown. Some possible hypotheses such as alterations in nucleotide metabolism leading to enhanced UDP-GlcNAc formation, an increased activity of OGT or an inhibition of OGA can be envisioned. We are currently investigating these possibilities to identify new molecular targets in the prevention of DR.

## Materials and Methods

### Animals

The care and experimental use of all animals in this study were in accordance with institutional guidelines and in compliance with the Association for Research in Vision and Ophthamology (ARVO) statement. All examinations were approved by the local ethics committee (Regierungspraesidium Kalsruhe). The NDPK-B^−/−^/Ang-2^+/−^, NDPK-B^−/−^/Ang-2^+/+^, NDPK-B^+/+^/Ang-2^+/−^ and NDPK-B^+/+^/Ang-2^+/+^ mice were generated by breeding the NDPK-B^+/−^/Ang-2^+/−^ and NDPK-B^+/−^/Ang-2^+/+^ mice. The transgenes of NDPK-B and Ang-2 were identified by genotyping as described previously by PCR analysis of ear DNA^10,12^.

### Retinal digestion

Frozen eyes were fixed in 4% Formalin for 48 h. The eyes were then placed under the loupe microscope and dissected. The retina was exposed by removing the lens and vitreous. Finally, the retina was extracted carefully. The isolated retina was incubated at 37 °C in water for 30 min, and subsequently incubated at 37 °C for 2.5 h in 3% trypsin dissolved in 0.2 M Tris-HCl buffer (pH 7.0). The retina was then transferred onto a glass object slide and carefully washed with dropping water until the pure retinal vasculature could be observed under the microscope. After the vasculature was dried on the object slide, it was stained with Period-Acid-Schiff (PAS).

### Retinal morphometry

The slides stained with PAS were observed under the microscope and photos of 40x magnification were taken. The numbers of pericytes and acellular capillaries were quantified. For quantification of pericytes, 10 microscopic fields with 40x magnification were randomly selected, and the pericytes as well as endothelial cells were counted. The cell numbers were recorded and normalized to the relative capillary density (numbers of cells per mm2 of capillary area). The capillary area was measured by the software AnalysisPro (Olympus Opticals, Hamburg, Germany). The acellular capillaries were quantified using an integration ocular with a grid of 100 squares. The numbers of squares containing acellular capillary segments were counted in 10 randomly selected microscopic fields of 40x magnification as described previously^12^.

### Isolation and culture of endothelial cells

Human umbilical vein endothelial cells (HUVECs) were obtained from umbilical cords of healthy newborns with the informed consent of their mothers. The use of HUVECs was approved by the local medical ethics committee (Medical faculty Mannheim, University of Heidelberg, Germany). All methods used were performed in accordance with the relevant guidelines and regulations of the ethics committee. Isolation and culture of HUVECs were described previously^9^. HUVECs were isolated by dispase digestion and cultured in endothelial cell growth complete medium (ECGM, Promocell) with 10% FCS on 1% gelatin-coated culture flasks. Cells until passage 3 were used for the experiments.

### Cell transfection

For transfection, HUVECs were seeded into a 12-well plate, cultured in ECGM containing 10% FCS overnight to allow them to reach 70% confluence. Then, gene knockdown was performed using lipofectamine (Life Technologies, Darmstadt, Germany) according to the manufacturer’s protocol. Specific siRNA and suitable scrambled siRNA as control (Eurofins MWG, Operon, Ebersberg, Germany) were used. The scrambled siRNA: AAC UGG UUG ACU ACA AGU CUU; the NDPK-B-specific siRNA: AGG UAG UGU AAU CGC CUU G; FoxO1-specific siRNA: ATG GTT CTA ATT TCC AGA TAA; the Ang-2-specific siRNA: GAA UUA GGG AAU GUU AAC GTG. Four hours after transfection, the cells were supplemented with ECGM containing 10% FCS, and cultured overnight. After incubation in ECGM containing 0.5% FCS for 24 h, the cells were subjected to O-GlcNAc inhibitor BADGP (2 mM) or Ac4–5S–GlcNAc (50 µM) diluted in ECGM containing 0.5% FCS for 24 h. Analyses were conducted at 48 and 72 h after transfection.

### Subcellular fractionation

For subcellular fractionation, HUVECs were transfected and cultured in 10 cm dish. Cells were scraped in PBS and lysed with homogenizer (POLYTRON® PT 1200B, KINEMATICA AG, Luzern, Switzerland). The cell homogenates were centrifuged at 200 g for 5 min at 4 °C, The nuclear fraction was precipitated in pellet while the cytosolic fraction was still soluble in supernatant. The cytosolic fraction was extracted further by centrifugation at 40000 g for 20 min at 4 °C. The pellet of cytosolic fraction was discarded and the supernatant (pure cytosolic fraction) was concentrated to 120 μl by Amicon® Ultra-0.5 Centrifugal Filter Devices (Merck Chemicals GmbH, Darmstadt, Germany). The concentrated purified cytosolic protein was mixed with 4x Laemmli sample buffer. In parallel, the nuclear fraction was re-suspended in 1.5 ml cold PBS, and further purified by homogenization and centrifugation at 40000 g for 20 min at 4 °C. After purification, the pellet of nuclear fraction was dissolved in 120 μl PBS and 40 μl 4x Laemmli sample buffer. Both the purified nuclear fraction and cytosolic fraction were heated for protein denaturation at 95 °C for 5 min and applied for immunoblotting.

### Immunoblotting

Western blot was performed using proteins extracted with RIPA buffer (50 mM Tris-HCl, pH7.4, 150 mM NaCl, 1 mM dithiothreitol, 1% Triton X-100, 1% sodium deoxycholate). The proteins were separated by SDS-PAGE and electrically transferred onto nitrocellulose membranes. After blocking with Roti-block (Roth, Karlsruhe, Germany), membranes were incubated with primary antibodies overnight. Immune complexes were incubated with corresponding secondary antibodies and visualized using a chemiluminescent peroxidase substrate (Roche, Mannheim, Germany; or Thermo Scientific, Rockford, USA). Protein expression was quantified using Image J (NIH, USA). Specific primary antibodies used were mouse-anti-NDPK-B (MC-412; Kamiya, Seattle, USA), rabbit-anti-FoxO1 (Cell Signaling, Leiden, Netherlands), rabbit-anti-Phospho-FoxO1 (Ser319) (Abcam, Cambridge, UK), goat-anti-Ang-2 (Santa Cruz, Heidelberg, Germany), mouse-anti-O-GlcNAc (Abcam); rabbit-anti-Akt (Cell Signaling), rabbit-anti-Phospho-SAPK/JNK (Thr183/Tyr185) (Cell Signaling), rabbit-anti-Phospho-c-Jun (Ser63) (Cell Signaling), rabbit-anti-Phospho-p38 MAPK (Thr180/Tyr182) (Cell Signaling), rabbit-anti-Histone H1 (Santa Cruz), mouse-anti-GAPDH (Meridian/Biodesign, Tebu-bio, Offenbach, Germany) and mouse-anti-γ-tubulin (Sigma-Aldrich, Munich, Germany). The corresponding secondary antibodies were rabbit anti-mouse peroxidase (Sigma-Aldrich), rabbit anti-goat peroxidase (Sigma-Aldrich) and goat anti-rabbit peroxidase (Sigma-Aldrich).

### Immunofluorescence

For immunofluorescence, HUVECs were seeded on gelatin-coated glass cover slips plated in 24-well plates and transfected with corresponding siRNA amount as described above. The cells were grown overnight in ECGM with 10% FCS. After incubation in 0.5% FCS for 24 h, the cells were washed with PBS and fixed with 4% formaldehyde for 10 min at room temperature. Then, cells were incubated in the blocking and permeabilization buffer containing 2.5% BSA and 0.3% Triton X-100. Subsequently, the cells were incubated with primary antibodies overnight at 4 °C. After washing with PBS, cells were incubated with secondary antibodies conjugated with FITC or TRITC for 1 h at room temperature. Finally, the slides were washed and mounted with mowiol (Calbiochem, Germany). The primary antibodies were rabbit-anti-FoxO1 (Cell Signaling), goat-anti-Ang-2 (Santa Cruz) and mouse-anti-O-GlcNAc (Abcam). The secondary antibodies were swine anti-rabbit FITC (DakoCytomation), swine anti-rabbit TRITC (DakoCytomation, Glostrup, Denmark), goat anti-mouse-FITC (Sigma-Aldrich) and Donkey anti-goat FITC (Acris, OriGene Europe, Herford, Germany). Photos were taken by confocal laser scanning microscopy (Leica Microsystems, Germany). Quantification of the protein expression in immunofluorescence was performed using Image J (NIH, USA).

### Pull down assay

HUVECs were transfected and cultured on 10 cm dish to 100% confluency. The cells were washed with ice-cold PBS three times and lysed by scraping with 500 μl lysis buffer (50 mM Tris-HCl pH 7.4, 100 mM NaCl, 1%TritonX-100, 1 tablet/10 ml protease inhibitor, Roche). The lysate was centrifuged at 13000 g for 15 min at 4 °C. Wheat-germ agglutinin (WGA) - conjugated agarose beads (Vector Laboratories, Biozol, Eching, Germany) were prepared according to manufacturer’s instruction. The supernatant was collected and mixed with the prepared beads, then incubated on a rotator at 4 °C overnight. After incubation, the beads were collected by centrifugation (200 g, 2 min), and washed three times with washing buffer. After washing, the beads were dissolved in 60 μl of 1× Laemmli buffer, and heated to denature the binding protein. The proteins were separated by SDS-PAGE gel electrophoresis and immunoblotted as described before with antibodies specific for O-GlcNAc, FoxO1 and Tubulin.

### Co-immunoprecipitation assays

The cell lysate was prepared as described in pull down assay. FoxO1 antibody (cell signaling), O-GlcNAc antibody (cell signaling) and Protein A beads (nProtein A SepharoseTM 4 Fast flow, GE Healthcare Europe GmbH, Freiburg, Germany) were employed to precipitate the O-GlcNAcylated FoxO1. Protein A beads were washed by RIPA buffer three times before use. In order to reduce the unspecific binding, cell lysate was incubated with the Protein A beads for 2 h, and collected by centrifugation (200 g, 2 min). Treated cell lysate was incubated with 10 μg FoxO1 antibody (Cell Signaling) (for FoxO1 Co-IP), 8 μg O-GlcNAc antibody (Cell Signaling) (for O-GlcNAc Co-IP), respectively on a rotator at 4 °C overnight. After incubation, the antibody-protein complexes were incubated with Protein A beads on a rotator (4 °C) overnight. Finally, the antibody-protein complexes which bind to Protein A beads were collected by centrifugation (200 g, 2 min). After washing, the beads which bind with the antibody-protein complex were dissolved in 60 μl of 1x Laemmli buffer and heated for 5 min at 95 °C to denature the protein. The samples were immunoblotted as described before with antibodies specific for FoxO1, O-GlcNAc and Tubulin. To investigate acetylation and ubiquitination, FoxO1 antibody (Cell signaling) was used to precipitate FoxO1. The precipitates were later probed with antibodies against Ubiquitin (Abcam) and Acetyl-Lysine (Cell signaling).

### Quantitative real time PCR

Quantitative PCR was performed as described previously^13^. All primers and MGB probes labelled with FAM for amplification were purchased from Applied Biosystems: FoxO1 Hs01054576_m1, Actin Hs99999903_m1. Expression of genes was analysed by the 2−ΔΔCT method using ß-actin as a reference gene.

### Statistical analysis

Data are presented as mean ± SEM, unless otherwise stated. Analysis of Variance (ANOVA) with Bonferroni post-test or unpaired student’s t-test with and without Welch’s correction was performed using GraphPad Prism 7 (GraphPad Software, La Jolla, CA, USA). p values < 0.05 were considered statistically significant.

### Data availability

The datasets generated during and/or analysed during the current study are available from the corresponding author on reasonable request.

## Electronic supplementary material

Supplementary figures
