# Supplementary material for: O-GlcNAcylation of FoxO1 mediates nucleoside diphosphate kinase B deficiency induced endothelial damage
Source: Sci Rep. 2018 Jul 12;8:10581. doi: 10.1038/s41598-018-28892-y (PMC6043576; doi:10.1038/s41598-018-28892-y)
Supplement: Supplementary file 1 — Supplementary figures [file 41598_2018_28892_MOESM1_ESM.pdf]

O-GlcNAcylation of FoxO1 mediates nucleoside diphosphate kinase B deficiency induced endothelial damage

Shenliang Shan[1,*], Anupriya Chatterjee[1,*], Yi Qiu[1], Hans-Peter Hammes[2], Thomas Wieland[1,3], Yuxi Feng[1]

[1] Experimental Pharmacology Mannheim (EPM), European Center of Angioscience, Medical Faculty Mannheim, Heidelberg University, Mannheim, Germany
[2] 5th Medical Clinic, Medical Faculty Mannheim, Heidelberg University, Mannheim, Germany
[3] DZHK (German Centre for Cardiovascular Research), partner site Heidelberg/Mannheim, Germany

* S.S and A.C share first authorship

Correspondence to:
Dr. Yuxi Feng, Experimental Pharmacology Mannheim (EPM), European Center of Angioscience, Medical Faculty Mannheim, Heidelberg University, Mannheim, Ludolf-Krehl-Str. 13-17, Tridomus C, 68167 Mannheim, Germany
Phone: +49-621-383-71762
Fax: +49-621-383-71751,
E-mail: yuxi.feng@medma.uni-heidelberg.de

Running Title: Protein GlcNAcylation under NDPK-B deficiency
Keywords: Angiopoietin 2, FoxO1, O-GlcNAc, NDPK-B, Endothelial

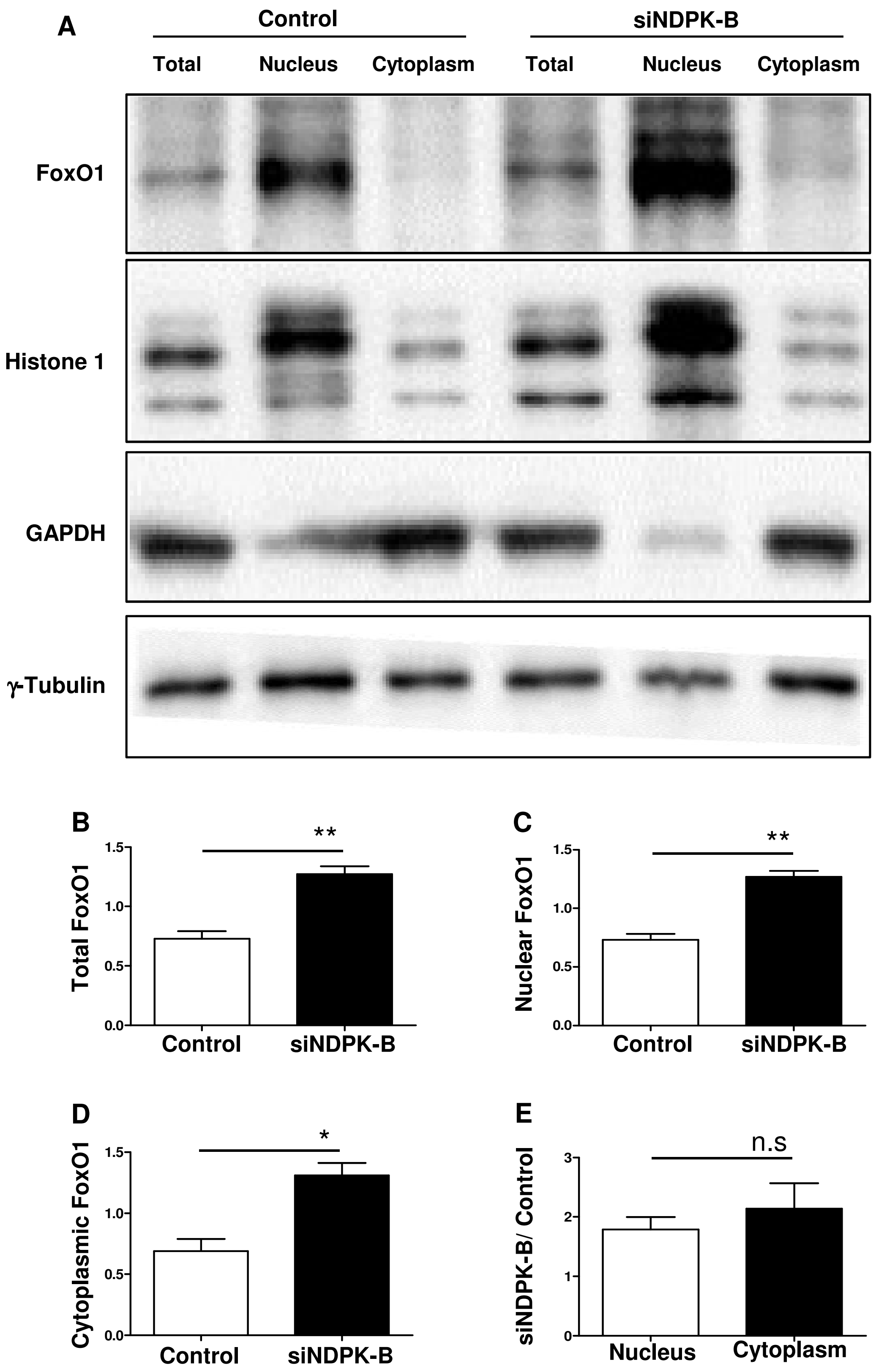
A
Control
siNDPK-B
Total
Nucleus
Cytoplasm
Total
Nucleus
Cytoplasm
FoxO1
Histone 1
GAPDH
γ-Tubulin
B
Total FoxO1
**
1.5
1.0
0.5
0.0
Control
siNDPK-B
C
Nuclear FoxO1
**
1.5
1.0
0.5
0.0
Control
siNDPK-B
D
Cytoplasmic FoxO1
*
1.5
1.0
0.5
0.0
Control
siNDPK-B
E
siNDPK-B/ Control
n.s
3
2
1
0
Nucleus
Cytoplasm

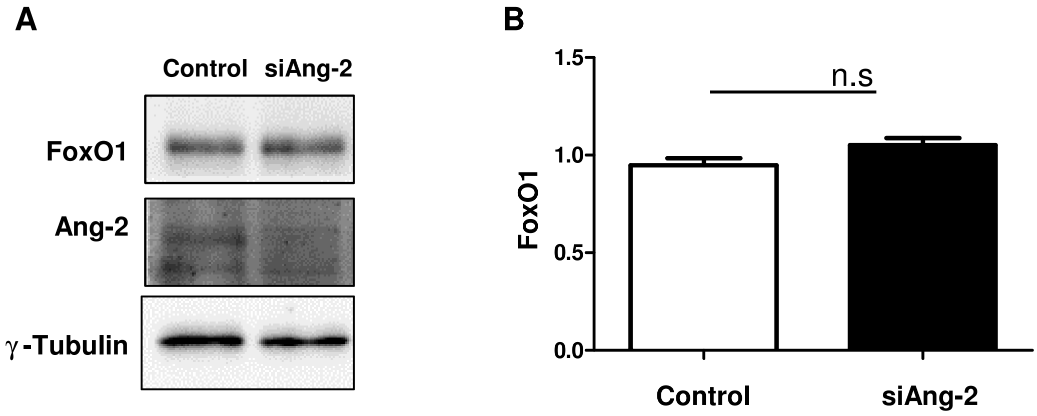
A
Control
siAng-2
FoxO1
Ang-2
γ-Tubulin
B
1.5
1.0
0.5
0.0
FoxO1
n.s
Control
siAng-2

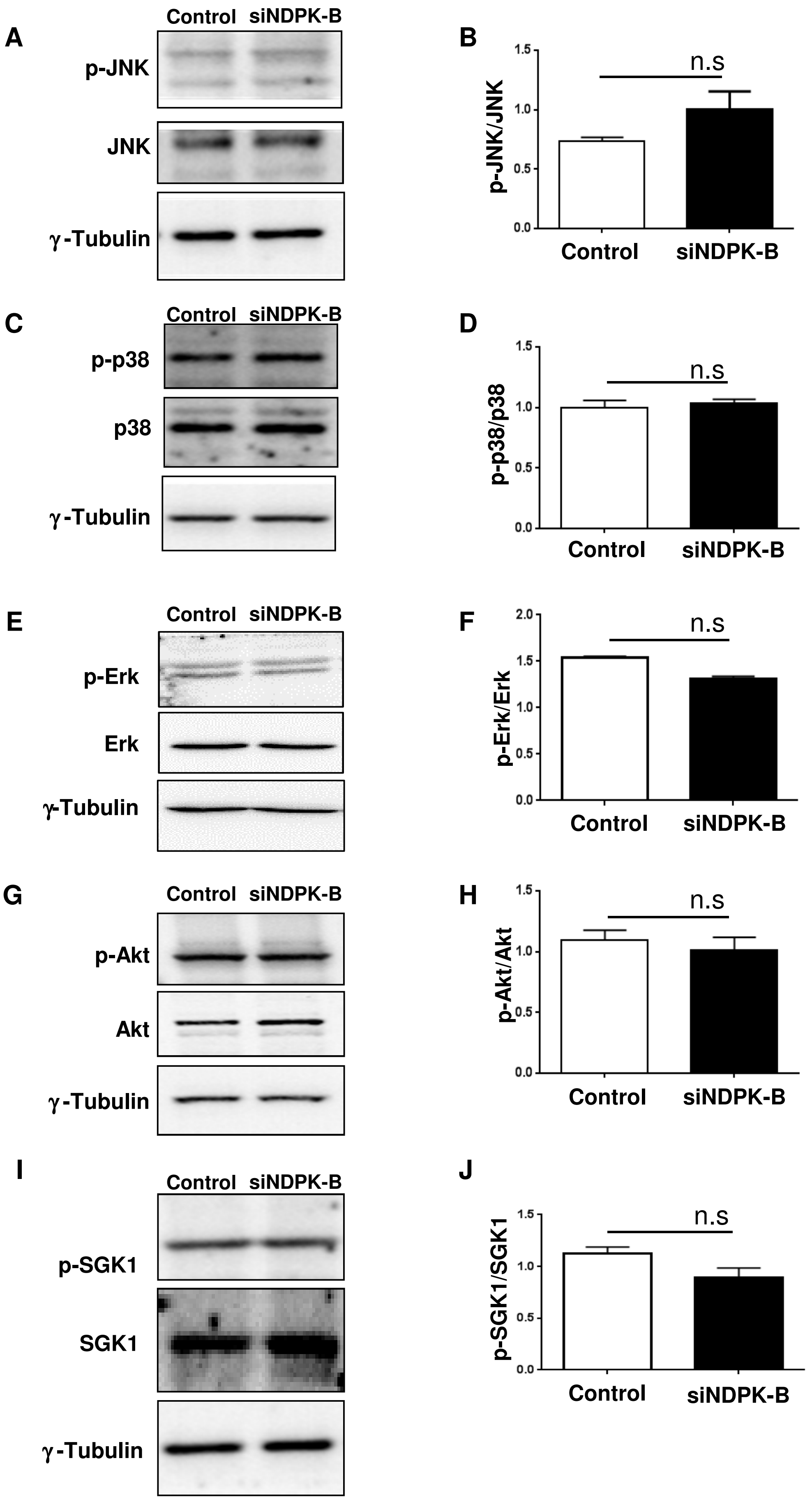
A
Control
siNDPK-B
p-JNK
JNK
γ-Tubulin
B
p-JNK/JNK
n.s
C
p-p38
p38
D
p-p38/p38
E
p-Erk
Erk
F
p-Erk/Erk
G
p-Akt
Akt
H
p-Akt/Akt
I
p-SGK1
SGK1
J
p-SGK1/SGK1

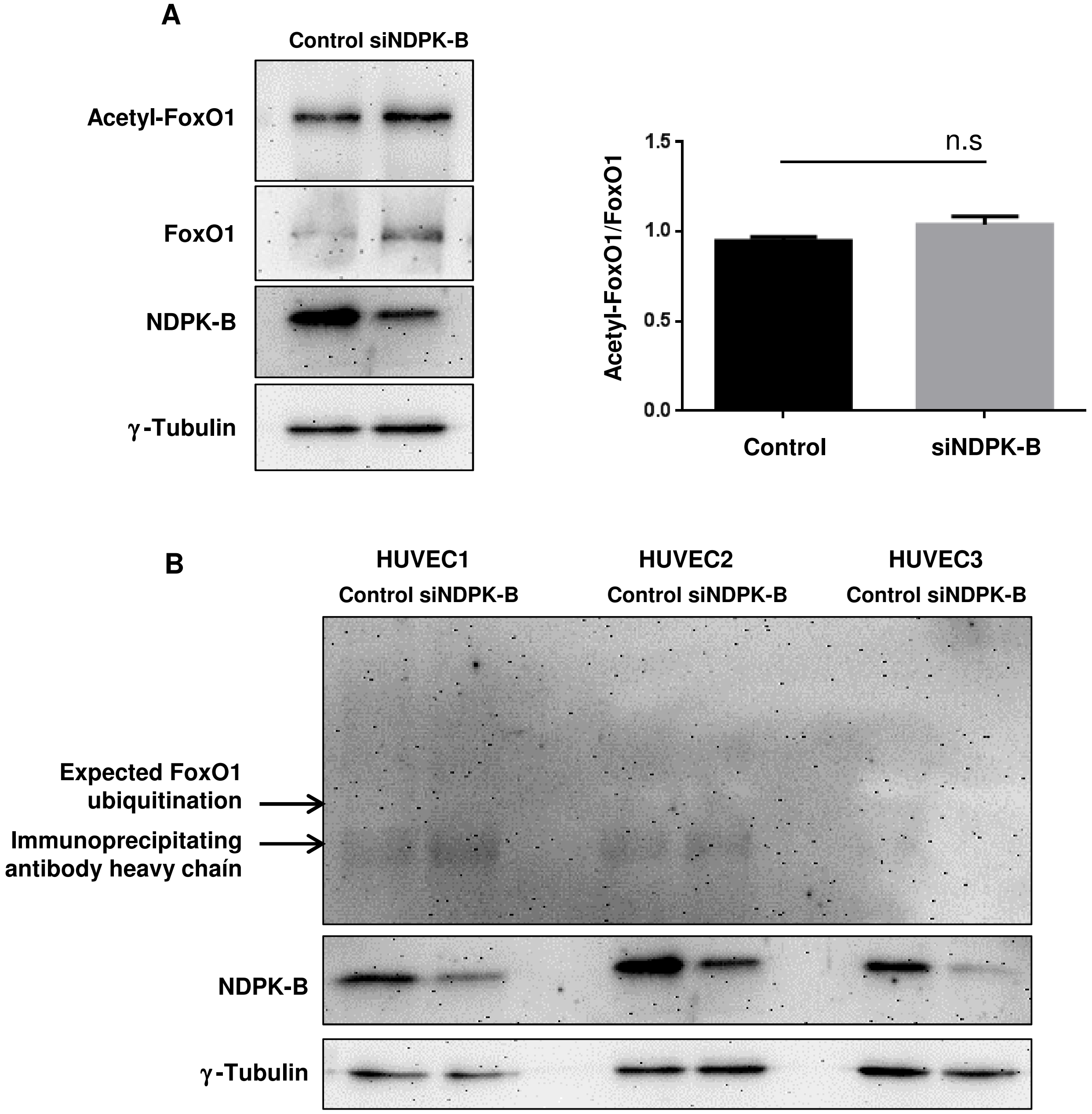
A
Control siNDPK-B
Acetyl-FoxO1
FoxO1
NDPK-B
γ-Tubulin
n.s
1.5
1.0
0.5
0.0
Acetyl-FoxO1/FoxO1
Control
siNDPK-B
B
HUVEC1
HUVEC2
HUVEC3
Control siNDPK-B
Control siNDPK-B
Control siNDPK-B
Expected FoxO1 ubiquitination
Immunoprecipitating antibody heavy chain
NDPK-B
γ-Tubulin

Supplementary information

O-GlcNAcylation of FoxO1 mediates Nucleoside diphosphate kinase B deficiency induced endothelial damage

Shenliang Shan, Anupriya Chatterjee, Yi Qiu, Hans-Peter Hammes, Thomas Wieland, Yuxi Feng

Supplementary Fig. S1: Depletion of NDPK-B does not impact the translocation of FoxO1 between the nucleus and the cytoplasm in ECs.

The total cell lysate, nuclear fraction and cytoplasmic fraction of cell lysate were probed with antibodies against FoxO1, NDPK-B, γ-tubulin (loading control for total cell lysate), Histone1 (loading control for nuclear fraction), GAPDH (loading control for cytoplasmic fraction). **A**: Representative immunoblots of FoxO1, Histone1, GAPDH and γ-tubulin. **B-E**: Quantifications of total FoxO1 (**B**), nuclear FoxO1 (**C**), cytoplasmic FoxO1 (**D**), comparison of nuclear and cytoplasmic FoxO1 in NDPK-B depleted and control cells (**E**) (n=5, *$p<0.05$, **$p<0.01$).

Supplementary Fig. S2: Ang-2 does not regulate FoxO1 in ECs.

Ang-2 deficient EC whole cell lysates were probed with antibodies against Ang-2, FoxO1 and γ-tubulin in Western blot. **A**: Representative immunoblots of FoxO1, Ang-2 and γ-tubulin (loading control). **B**: Quantification of FoxO1 in Ang-2 depleted ECs (normalized to γ-tubulin, n=3).

Supplementary Fig. S3: FoxO1 signaling in NDPK-B depleted ECs is Akt/SGK/MAPK independent.

NDPK-B deficient EC whole cell lysates were probed with antibodies against p-JNK, JNK, p-p38, p38, p-Erk, Erk, p-Akt, Akt, p-SGK, SGK and γ-tubulin in Western blot (n=3).

Supplementary Fig. S4: NDPK-B deficiency does not alter FoxO1 acetylation and ubiquitination.

Three separate isolations of ECs (HUVEC1, HUVEC2 and HUVEC3) were subjected to NDPK-B knockdown (A,B) followed by FoxO1 immunoprecipitation. NDPK-B depletion was verified by immunoblot in the cell lysate. γ-tubulin was used as loading control. The precipitates were subsequently probed with anti-acetyl lysine antibody (A, n=3; n.s: non-significant) and anti-ubiquitin antibody. Ubiquitination of FoxO1 was not detectable in any of individual HUVEC isolation (B).
